# Computational FEM Model,* Phantom* and* Ex Vivo* Swine Breast Validation of an Optimized Double-Slot Microcoaxial Antenna Designed for Minimally Invasive Breast Tumor Ablation: Theoretical and Experimental Comparison of Temperature, Size of Lesion, and SWR, Preliminary Data

**DOI:** 10.1155/2017/1562869

**Published:** 2017-12-10

**Authors:** Geshel David Guerrero López, Mario Francisco Jesús Cepeda Rubio, José Irving Hernández Jácquez, Arturo Vera Hernandez, Lorenzo Leija Salas, Francisco Valdés Perezgasga, Francisco Flores García

**Affiliations:** ^1^División de Estudios de Posgrado e Investigación, Tecnológico Nacional de México, Instituto Tecnológico de la Laguna, Blvd. Revolución, Esq. Calzad. Instituto Tecnológico de la Laguna, Zona Centro, 27000 Torreón, COAH, Mexico; ^2^Sección de Bioelectrónica, Centro de Investigación y de Estudios Avanzados del Instituto Politécnico Nacional, Ciudad de México, Mexico

## Abstract

Malignant neoplasms are one of the principal world health concerns and breast cancer is the most common type of cancer in women. Advances in cancer detection technologies allow treating it in early stages; however, it is necessary to develop treatments which carry fewer complications and aesthetic repercussions. This work presents a feasibility study for the use of microwave ablation as a novel technique for breast cancer treatment. A microwave applicator design is also being proposed for this purpose. The coupling of the designed antenna was predicted with computer simulation. The standing wave ratio obtained through simulation was 1.87 and the result of experimental validation was 1.04. The optimized antenna has an optimal coupling (SWR = 1.04) so ablation temperatures can be achieved in a relatively short time using low power. Varying the time and power, the heating pattern can be changed to treat different tumors. However, as some discrepancies are still present, a deeper study of the dielectric properties and their variation with temperature is required.

## 1. Introduction

The most frequent reported types of cancer worldwide (ordered by number of deaths) are lung, stomach, liver, colon-rectum, esophagus, and prostate, in the case of males, and breast, lung, stomach, colon-rectum, and cervix, in the case of females. Breast cancer is one of the most common types of cancer in women [1, 2], so we have focused our research on its minimally invasive treatment. Surgery is the conventional treatment for this type of cancer [3].

The present work proposes a new technique for its minimally invasive treatment by using microwave ablation (MWA). Ablation using microwave energy is an encouraging method because it can heat breast carcinoma (high water content tissue) more than adipose or breast glandular healthy tissue (low water content tissue); additionally, it can be used to treat patients that are not candidates for surgery like anesthetic risk. MWA presents advantages over other therapies [4–6] which make it more attractive to treat breast tumors; however, it is important to mention that to the best of our knowledge there is only one reported clinical trial of breast cancer treatment that uses MWA in literature [7]. Some of the benefits of MWA are an improved convection profile, higher constant intratumoral temperatures, faster ablation times, and the possibility of using multiple probes to treat multiple lesions simultaneously. In addition, the placement of grounding pads is not necessary; this reduces the risk of skin burns. Another advantage is that MWA creates larger ablation zones than other treatments with similar sized applicators [8]. Finally, the size and shape of the MWA zone may be more consistent and less dependent on the heat-sink effect from vascular structures in proximity of the lesion [9, 10].

Due to the increase in the use of mammography, the detection of breast cancer in initial stages has increased and this facilitates its treatment with noninvasive and minimally invasive techniques. These techniques are related to ablation technologies that can be classified as radio frequency ablation (RFA), MWA, laser photocoagulation, ultrasound ablation, and cryotherapy [11]. These treatments are a good alternative for patients who cannot be treated surgically.

MWA responds to the lower heating of adipose and glandular tissue compared to the heating of the breast carcinoma because of its higher water content [12]. The MWA occurs because of the movement of polar molecules in the tissue. Molecules try to align with the electric field generated and since the polarity of this field changes millions of times per second, they are constantly vibrating. This movement produces friction and the fiction results in tissue heating. The aim of MWA is the complete destruction of the cancer tissue and a safety margin of surrounding healthy tissue. The thermal destruction of tissue depends on the maximum temperature reached and the time of exposition; there are experimental studies supporting that 60 minutes at 43°C is tumoricidal, and the period of time required to kill tumor cells is halved for every degree increased above 43°C [13, 14]. In this work, 55°C isothermal contour was considered as ablation zone, since time needed to damage cells at this temperature is less than one minute [15]. A great variety of applicators have been proposed for their use in MWA; most of them are based on a coaxial structure. The coaxial applicator designs include the monopole, the dipole, and the slot applicators; they are usually covered with a polytetrafluoroethylene (PTFE) catheter in order to minimize the adhesion of the applicator to the ablation exposed (carbonized) tissue. A wide review of these applicators can be found in [16], although none of them have been applied to breast cancer treatment.

The dipole antenna is usually constructed with semirigid coaxial cable; for its design, three important regions should be considered. The first region is the distal tip of the antenna (usually referred to as extension) which is a metal segment of a specific length. The second region is a slot (commonly referred to as gap or junction) that acts as the effective source of electromagnetic wave propagation. Finally, the last region is the insertion depth of the antenna with a variable length. The dipole is an applicator of easy construction; however, the lesion produced by this antenna is highly dependent on the insertion depth.

The monopole is an applicator constructed with a semirigid coaxial cable in which the outer conductor is removed in order to leave an elongated inner conductor which is radially surrounded by dielectric materials. There are some variations of the monopole applicator, which depend on the finishing of the tip.

Finally, the slot antenna is one of the most popular designs for MWA. This is constructed by cutting a small ring slot in the outer conductor of a thin semirigid coaxial cable, and then the outer and inner conductor are short-circuited in the distal tip of the antenna. Some of the factors that modify the radiation pattern of this type of applicator are the width of the slot, the dielectric media surrounding the distal tip, and the length from the slot to the tip.

Since doing experimental validation for all these types of applicators is an extensive task, it turns useful to make use of computational models to face this problem. A numerical approach is an excellent way to solve complicated physical problems, considering that present hardware is not that expensive and it is powerful enough; thanks to this more researchers are using computational models to solve electromagnetic problems and component designs. There are several numerical approaches like Finite Difference Method that use finite differences to approximate the derivatives or the Finite Element Method (FEM) that subdivides a larger problem into smaller, simple parts, with a simple equation that models it. These equations are then assembled into a large system to model the entire problem.

There are several commercial software programs that use FEM like COMSOL, Abaqus, ANSYS, and SolidWorks. Among these software programs, the COMSOL uses electromagnetics and thermal libraries that permit the user to solve multiphysics problems making it possible to simulate the electromagnetic effect of the applicator and the thermal lesion produced in a single computation, which is the reason why it is used for this investigation.

In this work, an optimized double-slot antenna was constructed and computing modelled to be used in MWA therapy for breast cancer. The antenna was modelled by using the Finite Element Method and the Maxwell and bioheat equations. Then, the antenna was constructed by using a micro coaxial cable which was modified in order to have two slots. In both cases, the temperature distribution and the Standing Wave Ratio (SWR) were obtained. Experiments were carried out in breast mimicking phantom and* ex vivo* swine breast. The SWR and temperature distribution were compared in order to determine the feasibility of using this novel technique in breast cancer treatment.

## 2. Materials and Methods

### 2.1. Applicator Design

The applicator proposed here consisted of a micro coaxial cable with two 1 mm wide slots situated in the distal part of the antenna. The diameter of the applicator was approximately 2.2 mm; the outer conductor was made of copper; the dielectric was low loss PTFE and the inner conductor was silver plated copper wire (SPWC).  Table 1 shows dimension details and thermal properties of the antenna and of the breast tissue.

The geometrical parameters of the antenna were selected considering the effective wavelength in tumor tissue at 2.45 GHz (this frequency was selected considering that it belongs to ISM band and it is available worldwide). The wavelength was calculated by using the following equation:

(1) $$\begin{array}{c} \lambda_{eff}=\frac{c}{f\sqrt{\varepsilon_{r}\mu_{r}}}, \end{array}$$

where *c* represents the speed of light in free space (in m/s), *f* represents operating frequency of the generator (2.45 GHz), *ε*_*r*_ represents the relative permittivity of the tumor at operating frequency (59.385), and *μ*_*r*_ represents the magnetic relative permeability (1). The calculated wavelength for these values was 15.88 mm; however, since the tissue was heterogeneous, this value was used only as a reference.

**Figure 1:**
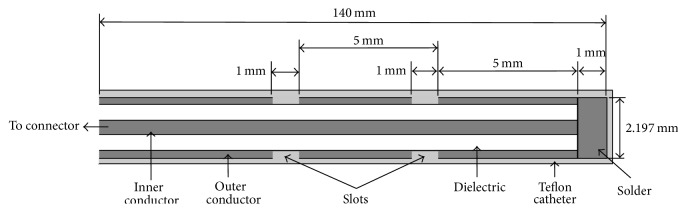
Axial view of the dimensions of the double-slot antenna used for MWA. The two 1 mm slots were separated by 5 mm; the total length of the applicator was 140 mm.

The applicator geometry is shown in Figure 1. Notice that the distance between the first slot and the tip and the distance between both slots are approximately 0.25*λ*_eff_.

### 2.2. The Finite Element Method

Many researchers have used mathematical models based on computer electromagnetics models for the applicator design. These methods are focused on the solution of electromagnetic fundamental equations like the Maxwell equations. There are three primary computer electromagnetics models: Finite Difference Method, Method of Moments, and FEM. The FEM can rapidly provide the users with solutions to multiple differential equations systems and therefore it satisfies the heat-transfer problems [19]. Moreover, it is a numerical technique that can be formulated as functional minimization. FEM involves dividing a complex geometry into small elements for a system of partial differential equations, evaluated at nodes or edges. The method is the formulation of solutions to the fundamental electromagnetic equations, known as Maxwell equations. However, in order to develop accurate models of the ablation process, the knowledge of tissue electromagnetic properties, like permittivity and conductivity and appropriate initial and boundary conditions, must be known.

### 2.3. Computer Model Definition

**Figure 2:**
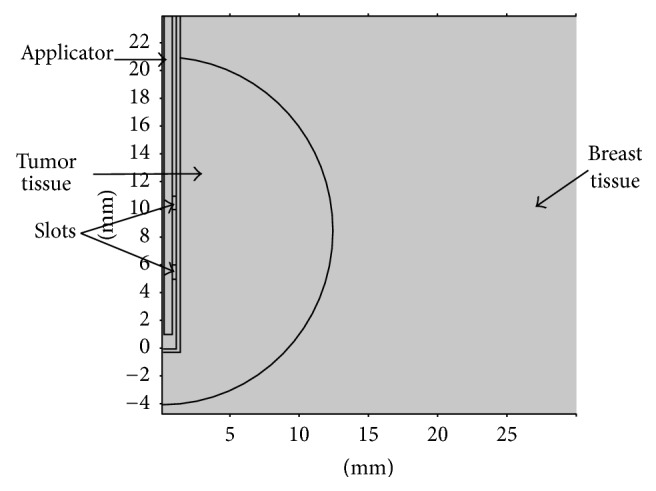
Axisymmetric view of the geometry used for the computer model. Tumor was represented as a semicircle surrounded by breast tissue; the slots were positioned inside the tumor. Units are in millimeters.

The simulations were carried out by using COMSOL Multiphysics™ 4.4 software (Comsol Inc., Burlington, MA). Since the model had an axial symmetry, a 2D axisymmetric model was used to minimize computing time. It was assumed for the model that the antenna was introduced into the neoplasm surrounded by breast tissue. The vertical axis was oriented along the longitudinal axis of the antenna and the horizontal axis was oriented along the radial direction as shown in Figure 2.

**Figure 3:**
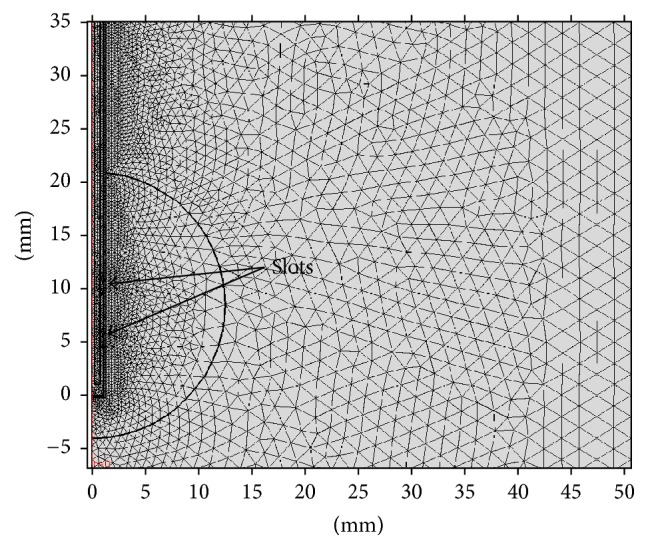
Mesh used for computational simulation; the mesh is finer near the slots of the applicator where maximum effect is expected.

The boundary conditions for the electromagnetic simulation are scattering boundary condition for the exterior boundaries, with the *z*-axis as axial symmetry; for the thermal simulation the boundary conditions are all exterior constant temperature (25°C) with the *z*-axis as axial symmetry. Finite Element Method (FEM) has been used to simulate the performance of microwave coaxial antennas. Linear solver performed FEM model constructing two-slot antenna geometry. Mesh settings presented on FEM model had 0.004 mm and 2 mm minimum and maximum element size, respectively; the maximum element size was chosen considering the minimum wave length present in the experiment (approximately 2.1 mm for tumor tissue at a frequency of 2.45 GHz); the model had 27 vertex elements, 2412 boundary elements, 12135 elements, and 42756 degrees of freedom solved.  Figure 3 shows the mesh for the simulation; it is finer near the slots where the maximum temperature is expected. Model solution took 25 seconds using 1.18 GB of physical memory and 1.42 GB of virtual memory. 4.0 GHz Intel Core i7 32 GB 1333 MHz DDR4 RAM PC was used to process the model.

For the evaluation of MWA performance, it was important to measure the frequency-dependent reflection coefficient and the specific absorption rate (SAR) pattern in the tissue. A reflection coefficient describes either the amplitude or the intensity of a reflected wave relative to an incident wave. This coefficient can be expressed logarithmically as

(2) $$\begin{array}{c} \Gamma \left(\;f\;\right)=10\times {log}_{10}\left(\;\frac{P_{r\left(f\right)}}{P_{in}}\;\right)\;\;\left(dB\right), \end{array}$$

where *P*_in_ is the input power and *P*_*r*_ represents the reflected power (both in Watts). SAR represents the amount of average power deposited per unit mass of tissue (W·kg^−1^) at any position; it can be expressed as

(3) $$\begin{array}{c} SAR=\frac{\sigma }{2\rho }{\left|\;\vec{E}\;\right|}^{2}\;\;\left(\text{W}\cdot {\text{kg}}^{-1}\right), \end{array}$$

where *σ* represent tissue conductivity (S·m^−1^), *ρ* is the tissue density (kg·m^−3^), and *E* represents the electric field (V·m^−1^). The absorption of microwave energy causes tissue temperature to rise; however, it does not determine the final tissue temperature distribution. Thermal effects should be considered and Pennes bioheat equation can be used for this purpose [20]:

(4) $$\begin{array}{c} \nabla \cdot \left(\;-k\nabla T\;\right)=\rho_{bl}C_{bl}\omega_{bl}\left(\;T_{bl}-T\;\right)+Q_{met}+Q_{ext}, \end{array}$$

where *k* represents tissue thermal conductivity (W·m^−1^·K^−1^), *ρ*_bl_ is the blood density (kg·m^−3^), *C*_bl_ represents the specific heat capacity of the blood (J·kg^−1^·K^−1^), *ω*_bl_ is the blood perfusion (kg·m^−3^·s^−1^), *T*_bl_ represents blood temperature (K), and *T* is the final temperature (K). The physical phenomena with the highest repercussion in the equation are microwave heating and tissue heat conduction. The temperature of the blood is approximately the same as body core temperature. Heat radiation (heat conduction) and metabolism heat generation are assumed to be minimal during MWA and are neglected. Also, in* ex vivo* samples *ω*_bl_ = 0 since there is no perfusion. For *Q*_ext_, microwave heating is considered; the microwave generates heat by resistive heating, and the rate of heat is proportional to the square of the applied electric field (*E*):

(5) $$\begin{array}{c} Q_{ext}=\sigma E^{2}. \end{array}$$

For the computational model, an antenna, constructed with a 50 Ω semirigid coaxial cable (Model UT-085, Micro-Coax, PA, US) [21], was used. The antenna was encased in a PTFE catheter in order to prevent adhesion of the antenna to desiccated ablated tissue. It was important to add the PTFE catheter to the simulation because, according to [18], it impacts on the SAR distribution and also on the heating pattern.

The conditions used in the simulation are shown in Table 2.

### 2.4. Model Validation

**Figure 4:**
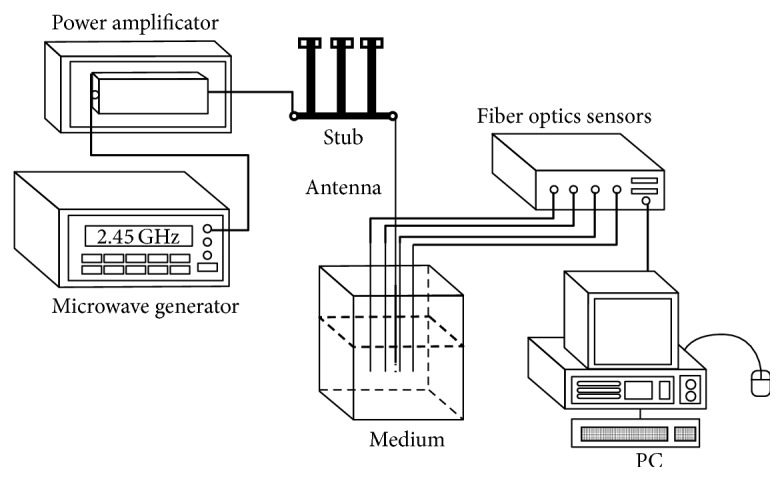
Setup for the experimental validation. A 2.45 GHz microwave was generated and amplified to the desired power level; then it was connected to the antenna through a stub. Sensors were used to measure temperature fiber optic.

In order to validate the performance of the simulated applicator, it was built by using a microcoaxial semirigid cable (UT-85) and a SMA connector.  Figure 4 shows the diagram for the experimental validation.

### 2.5. Electromagnetic Radiation System

In order to excite the applicator, a signal generator (Model SML03, Rohde & Schwarz, Munich, Germany) connected to an amplifier (Model 1164-BBM3Q6AHM, EMPOWER, NY, US) was used to deliver a 10 W signal for the applicator tests.

### 2.6. Standing Wave Ratio and Dielectric Properties Measurement

The Standing Wave Ratio (SWR) measurement was performed in two ways: with and without a coupling impedance network. The antenna was immersed in a phantom and was connected to a network analyzer (Model E5071B ENA, Agilent, CO, USA) to measure the reflection coefficient of the antenna. This equipment was also used to measure the dielectric properties of the breast tissue and the phantoms by the use of the 85071E materials measurement software (Model 5988-9472EN, Agilent, CO, USA) and 85070E dielectric open ended coaxial probe kit (Model 5989-0222EN, Agilent, CO, USA).

The impedance matching network consisted of a stub type tuner (Model 2612C2, Maury Microwave Corporation, CA, USA).

### 2.7. Phantom Design

A phantom is a mixture of different materials with similar dielectric properties to those of human tissue. The permittivity of tumor tissue is approximately tenfold higher than that of normal tissue [12].

**Figure 5:**
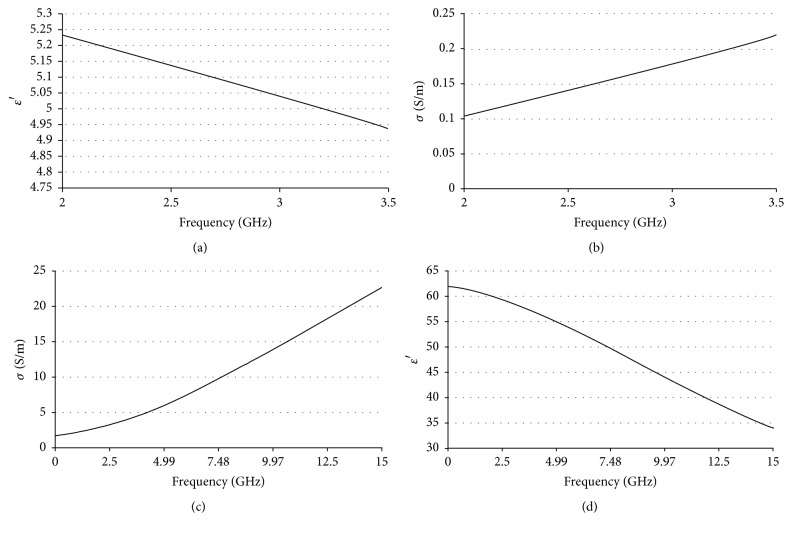
Breast relative permittivity (a) and conductivity (b) and tumor relative conductivity (c) and permittivity (d) obtained from [12]. It is important to notice that tumor properties were approximately tenfold higher than those of breast.

Previous works were considered in the design and construction of the phantom [12, 24]. Relative permittivity and conductivity are very important properties for the MWA work; therefore, they were measured after the phantom elaboration with the network analyzer.  Figure 5 shows these properties as a function of frequency.  Table 3 shows the values measured for a 2.45 GHz frequency.

For breast phantom elaboration, vegetable oil (150 ml), bidistilled water (50 ml), neutral detergent (30 ml), and agarose (4.5 g) were mixed and heated; breast tumor phantom was composed of bidistilled water (100 ml), ethanol (60 ml), sodium chloride (1 g), and agarose (1.5 g).

**Figure 6:**
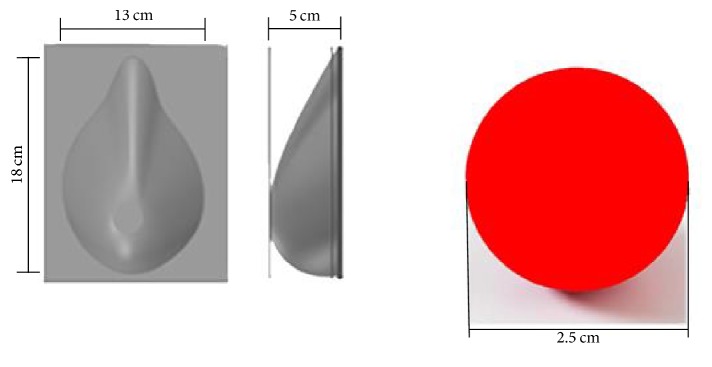
Molds used to fabricate breast and tumor phantoms. The size of the breast mold was the mean value of the measurements of ten 34B brassiere size volunteer women. The size for the cancer tissue mold corresponded to a tumor that had gone from T1 to T2.

As for the geometry used to prepare the phantom, a breast-shaped mold for breast tissue phantom and a sphere of 2.5 cm in diameter for the cancerous tissue phantom were used. The size of the breast mold was the mean value of the measurements of a group of ten volunteer women whose brassiere size was 34B. The sphere geometry was selected because it has axial symmetry, and the sphere size was chosen taking into account a tumor that had gone from T1 to T2, according to TNM Classification of Malignant Tumors [25]. These geometries are shown in Figure 6. Both phantoms are solids; after the elaboration of the tumor phantom the sphere was removed from the mold and then it was inserted in the breast phantom mixture before it solidified.

### 2.8. Validation Experiment

**Figure 7:**
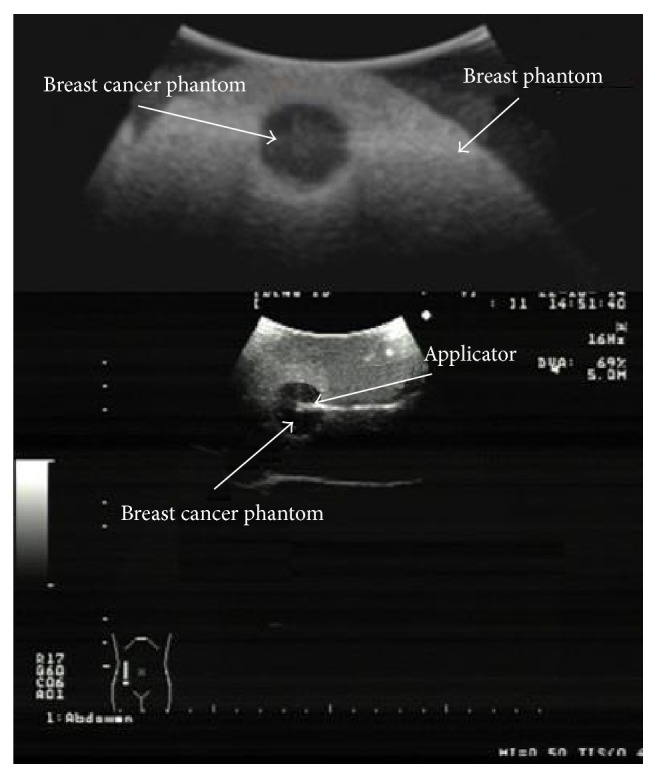
Positioning of the applicator. Image obtained through the ultrasonic scanner (model ProSound 6, Aloka, Switzerland). The antenna is shown at the center of breast cancer phantom.

**Figure 8:**
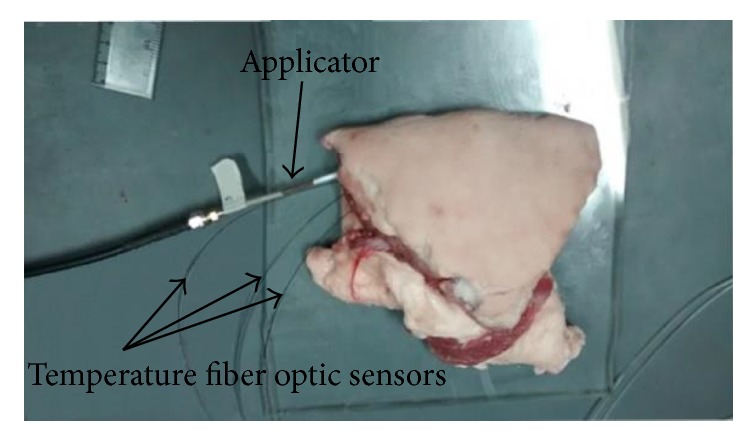
Model validation. Applicator and temperature sensors inserted into* ex vivo* swine breast tissue.

The applicator was inserted inside the tumor phantom which was surrounded by the breast phantom. An ultrasonic scanner (model ProSound 6, Aloka, Switzerland) was used along the process in order to verify that the position proposed in the computational model was achieved, Figure 7. Once the applicator was positioned, SWR measurements were made. For the validation of the temperature reached, the applicator was inserted into* ex vivo* swine breast tissue obtained from a local slaughterhouse. In order to place the applicator and temperature sensors, the tissue was half open.  Figure 8 shows the setup of this validation experiment.

**Figure 9:**
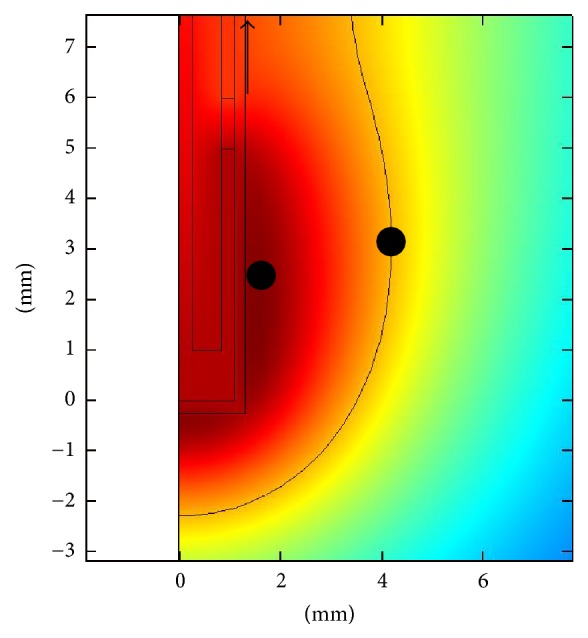
Points where temperature sensors were positioned in validation experiment. One in the hottest point, coordinates “1.6 mm, 2.6 mm,” one in the maximum radius of ablation (4.2 mm, 3 mm), and the last one close to tissue surface next to the applicator.

In order to measure the temperature inside the tissue, three temperature fiber optic sensors (STB Medical probe, Luxtron MAR'05, Lumasense, Santa Clara, CA, USA) were used. The temperature range of the probes was 0 to 120°C, their length was 1 m with a diameter of 0.5 mm, and the response time was 0.25 s. The sensors were non-electromagnetically interfering. They were connected to a thermometer (Model 3300, Luxtron, CA, US), with a resolution of 0.01°C, in order to monitor temperature and to store the data, via a RS-232 cable, in a personal computer. The sensors were placed one in the hottest point according to simulation (1.6 mm, 2.6 mm), one next to the applicator close to the tissue surface (1.32 mm, 100 mm), and the last one at a distance from the applicator equal to the maximum ablation radius (4.2 mm, 3 mm) as shown in Figure 9.

## 3. Results and Discussion

Successful ablation is considered when total destruction of the tumor is achieved. This destruction depends on the temperature reached on the tumor, which in turn is determined by the energy transfer and coupling between the antenna and the radiated media (phantom or tissue); right coupling is necessary to prevent backward heating. For these reasons, the most important parameters to consider in the design of the microcoaxial antenna are the maximum temperature reached, the SWR, and the ablation zone. These results are presented below, first for the computer simulation and afterwards for the experimental validation. Finally, a comparison of both is presented.

### 3.1. Computer Model Results

**Figure 10:**
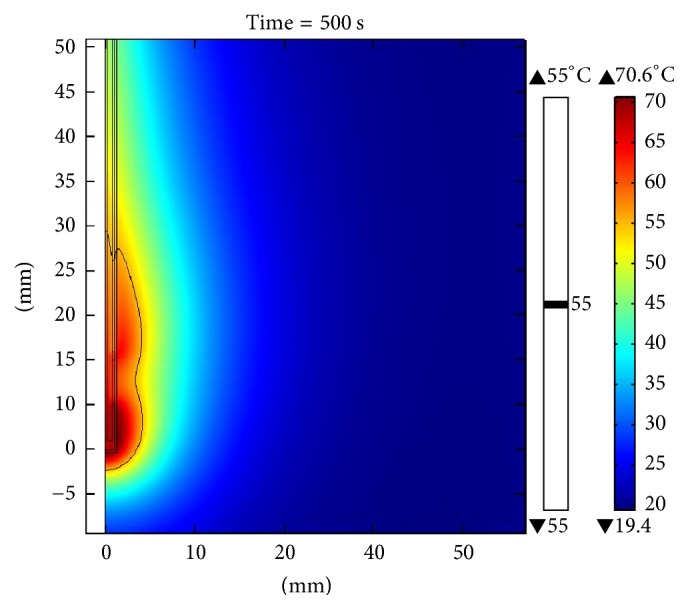
Temperature distribution for the simulation when the applicator was inserted in breast tissue. The power applied was 10 W; the maximum temperature reached, after 500 seconds, was 70.6°C.

**Figure 11:**
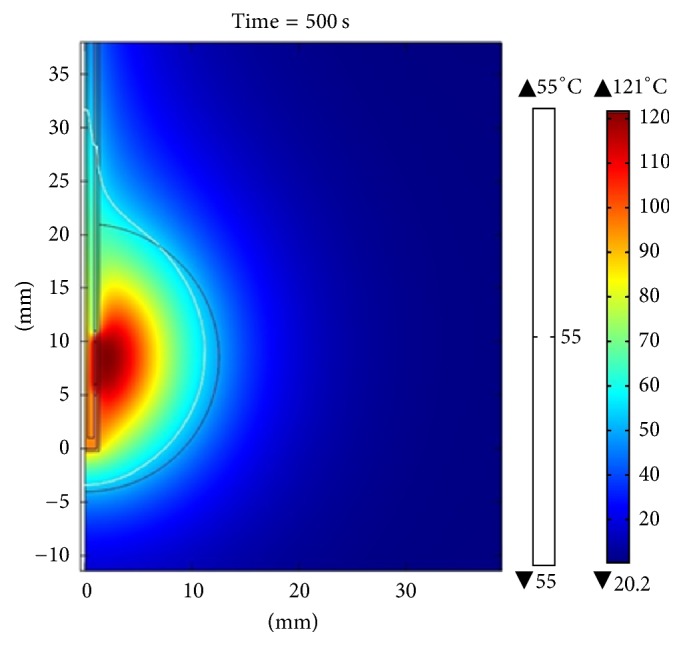
Temperature distribution for the simulation when the applicator was inserted in a spherical tumor of 12.5 mm in radius surrounded by breast tissue. The power applied was 10 W; the maximum temperature reached, after 500 seconds, was 110°C.

**Figure 12:**
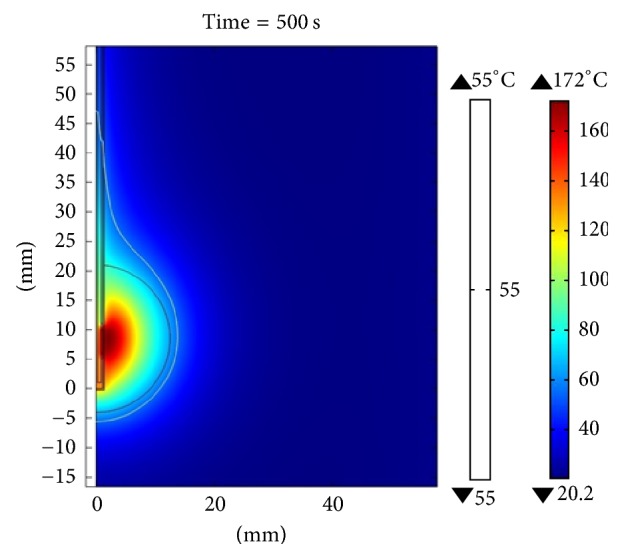
Temperature distribution for the simulation when the applicator was inserted in a spherical tumor of 12.5 mm in radius surrounded by breast tissue. The power applied was 15 W; the maximum temperature reached, after 500 seconds, was 155°C.

Figure 10 shows the distribution of the temperature obtained through the computer model when the applicator was inserted into the breast tissue. The maximum temperature reached after 500 seconds (time required for steady state) was 70.6°C for an applied power of 10 W. The maximum radius of the lesion was 4.2 mm (measured from the center of the applicator to the farthest radial point of the 55°C isothermal curve), and the SWR for the frequency of 2.45 GHz was 1.84.  Figure 11 shows the temperature distribution in a clinical situation where the applicator was inserted in a spherical tumor of 12.5 mm radius surrounded by breast tissue. The maximum temperature reached for a power of 10 W, after 500 seconds, was 110°C. The maximum radius of injury was 11.2 mm and the SWR for the frequency of 2.45 GHz was 1.87. It is important to note that, with a power of 10 W, the injury was not large enough to cover the entire tumor, but when therapy was performed with a power of 15 W, total tumor ablation was achieved. The maximum temperature reached was 155°C, after 500 seconds, as shown in Figure 12.

### 3.2. Experimental Validation

**Figure 13:**
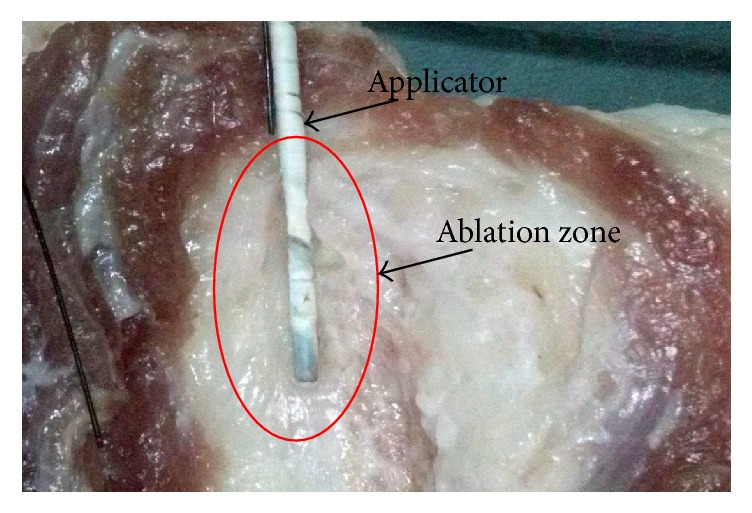
Injury produced by the applicator in* ex vivo* swine breast tissue.

For the experimental validation, the temperature measurement was carried out in* ex vivo* swine breast tissue. Additionally, the SWR measurement was performed in a tumor phantom surrounded by a breast phantom; in order to emulate the conditions of the simulation, experiments where performed in triplicate.  Figure 13 shows the lesion produced by the applicator inserted* ex vivo* into swine breast tissue. The maximum temperature reached for a power of 10 W was 90°C and the maximum radius of the lesion was 4.3 mm. Moreover, the SWR measured in tumor phantom surrounded by breast phantom for a frequency of 2.45 GHz was 1.04.

### 3.3. Comparison

**Figure 14:**
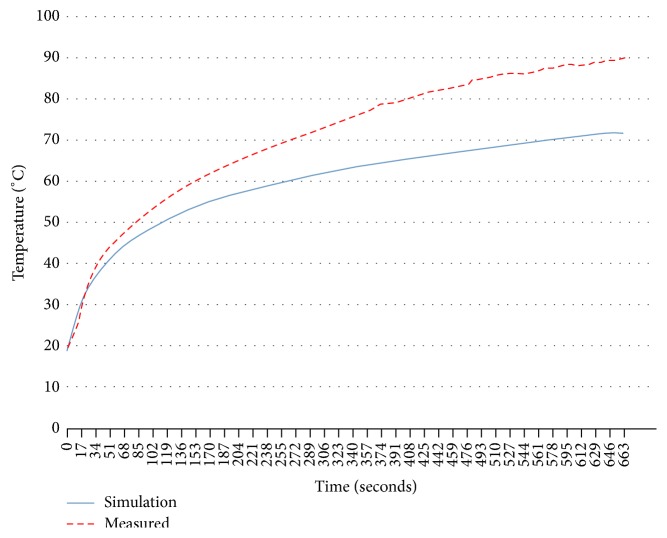
Temperature against time for two-slot applicator in* ex vivo* swine breast tissue.

**Figure 15:**
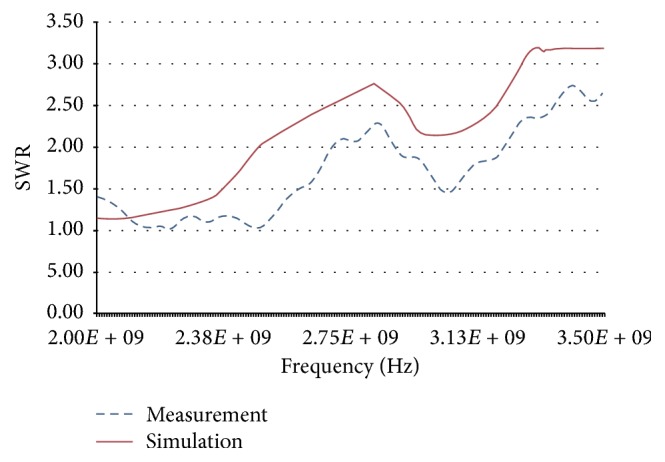
Comparison of SWR obtained through simulation (red) and experimental validation (blue).

As mentioned above, the three most important results were the maximum temperature reached, the diameter of the lesion, and the SWR. The maximum temperature obtained through simulation with a power level of 10 W and a time of 500 s in healthy breast tissue was 70.6°C, while the maximum temperature reached during the experimental validation in* ex vivo* swine breast tissue was 90°C (Figure 14 shows the temperature rise versus time, both for the simulation and the experimental validation). The maximum radius of the lesion was 4.2 mm and 4.3 mm for simulation and validation experiments, respectively. Finally, the SWR obtained in the simulation of the clinical situation (a tumor of 12.5 mm radius surrounded by breast tissue) for a frequency of 2.45 GHz was 1.87, while the SWR obtained in the experimental validation (sphere tumor phantom of 12.5 mm radius surrounded breast phantom) was 1.04.  Figure 15 shows the comparison of the SWR for the frequency band from 2 GHz to 3.5 GHz. A resume of these comparisons is shown in Table 4.

## 4. Conclusions

Microwave ablation by using double-slot microcoaxial antennas as applicators is a promising technique for cancer treatment. A feasibility study of the use of this technique for breast cancer treatment was carried out. The results specifically analyzed were temperature, size of lesion, and SWR, of a computational FEM model and physical models (breast phantom and* ex vivo* swine tissue).

The simulations demonstrated the possibility of obtaining ablation temperatures, that is, temperatures higher than 55°C. It was also observed that when the tumor was inside the breast tissue, the heat was focalized inside the tumor and higher temperatures were obtained. This can be explained due to the differences of dielectric properties between tumor and healthy breast tissues, which may be an advantage because cancerous tissue can be eliminated without affecting healthy tissue. It is important to mention that, by changing time and power, it was possible to change the size of the lesion caused by ablation; therefore, adjusting these two variables, it would be possible to treat tumors of different sizes.

Concerning the results obtained in physical models, phantom, and swine breast tissue, the possibility of reaching ablation temperatures was confirmed. The temperature obtained was 90°C for swine breast tissue, and the lesion was pear-shaped as in the computational model.

For the SWR, in the physical models a low value, 1.04, was obtained. This value approximated much more to the ideal value, 1.0, than that obtained from the simulation, which was 1.87. This result could be explained on the fact that the dielectric properties of tissue were not as constant and homogenous as they were considered for the simulation. It will also be important to consider that there are variations of dielectric properties from subject to subject, and it will be necessary to include a system to reduce the reflection of the applicator under clinical conditions. For this reason, it would be recommendable to determine the subject's tissue properties in order to run a computational model that determines the best position of the applicator.

However, it is still necessary to validate these results in* ex vivo* breast cancer tissue and* in vivo* laboratory tests before concluding if it is feasible to use this microcoaxial antenna for breast cancer treatment.

## Figures and Tables

**Table 1:** Dimensions and properties of the materials used for the FEM modelling of the double-slot microcoaxial antenna.

Parameter	Value	Reference
Central conductor diameter	0.51 ± 0.0127 mm	[17]
Dielectric diameter	1.68 ± 0.0254 mm	[17]
External conductor diameter	2.197 ± 0.0254 mm	[17]
Catheter diameter	2.64 ± 0.03 mm	—

Material	Relative permittivity	Reference

Coaxial cable internal dielectric	2.03	[18]
Catheter	2.60	[16]

**Table 2:** Conditions used for simulation of MTA treatment on breast and tumor phantom.

Parameter	Value
Space dimension	Axial symmetry 2D
Heat-transfer module	Time-dependent analysis
RF module	Frequency domain analysis
Boundary RF module	Setting
*z* axis	Axial symmetry
Port mode	Coaxial
All others	Impedance (Air)
Boundary heat-transfer module	Setting
All exterior	Constant temperature (25°C)
Mesh	Value
Number of vertex elements	27
Number of boundary elements	2412
Number of elements	12135
Breast thermal conductivity	0.42 W/m °K [22]
Breast cancer thermal conductivity	0.50 W/m °K [22]
Blood density	920 Kg/m^3^ [22]
Blood heat capacity	3639 J/Kg/°K [23]
Blood perfusion rate	0.0036 s^−1^ [23]

**Table 3:** Dielectric properties of breast and tumor tissue at 2.45 GHz.

Tissue	Conductivity (s/m)	Relative permittivity
Tumor	3.156	59.385
Breast	0.137	5.1467

**Table 4:** Comparison of temperatures (after 500 seconds), SWR, and lesion radius between simulation and experiment.

Medium	Temperature (°C)		SWR		Radius (mm)	
	Simulation	Experiment	Simulation	Experiment	Simulation	Experiment
Breast	70.6	85.43	1.84	—	5	4.1
Breast tumor (10 W)	110	—	1.87	1.04	11.17	—
Breast tumor (15 W)	155	—	1.87	1.04	13.86	—

## Abbreviations

RFA: Radio frequency ablation
MWA: Microwave ablation
PTFE: Polytetrafluoroethylene
FEM: Finite Element Method
SWR: Standing wave ratio.
